# DiGS: Depth-Initialized Gaussian Splatting for Single-Object Reconstruction

**DOI:** 10.3390/jimaging12050183

**Published:** 2026-04-24

**Authors:** Jacopo Meglioraldi, Pasquale Cascarano, Gustavo Marfia

**Affiliations:** Department of the Arts, University of Bologna, Via Barberia 4, 40123 Bologna, Italy; jacopo.meglioraldi@studio.unibo.it (J.M.); gustavo.marfia@unibo.it (G.M.)

**Keywords:** gaussian splatting, 3D reconstruction, computer vision, depth map, RGB, image processing

## Abstract

Gaussian Splatting is a state-of-the-art technique for 3D reconstruction. In this paper, we investigate how different initialization strategies influence the optimization process within the Gaussian Splatting framework, showing that more accurate initial point clouds can greatly influence the quality of object reconstruction. We introduce the Depth-initialized Gaussian Splatting (DiGS) approach, a pipeline that leverages depth-based initialization. By incorporating depth data from a calibrated stereo camera setup, the proposed method significantly enhances model performance, particularly during the early optimization stages. DiGS is particularly effective for reconstructing isolated single objects and improving the recovery of fine-grained details. Several tests on synthetic and real-world datasets confirm the effectiveness of the proposed pipeline. To evaluate our approach, we employ objective metrics and a user study involving 20 participants to assess with human perception the quality of the proposed approach.

## 1. Introduction

The capability to digitally replicate real-world objects and environments has become increasingly important in many fields like robotics, entertainment, cultural heritage, and medicine [1,2,3,4,5], and in the last decade, researchers have been focusing on advancing 3D reconstruction techniques [6,7]. Accurate 3D digital representations enable detailed analysis, efficient synthesis, and precise manipulation of their physical counterparts for simulation, training, and remote collaboration [6,8,9,10].

In robotics, 3D digital models enable autonomous manipulation and navigation through precise reconstruction of both objects and robotic arms, essential for planning adaptive motion sequences and simulating contact points and grasping [11,12,13]. Real-time 3D mapping and obstacle detection further support safe navigation, particularly in human-shared environments [14,15,16]. In cultural heritage, 3D scanning facilitates preservation, non-invasive analysis, and enhanced public access, while museums employ these technologies to create immersive exhibits that enrich storytelling [17,18,19,20]. The entertainment sector employs advanced 3D modeling and rendering to enhance the stages of realism in visual effects as well as interactive experiences [21,22,23,24,25,26,27]. In medicine, 3D reconstruction supports medical training and diagnosis with detailed anatomical models enhancing surgical precision [28,29,30,31,32].

Standard 3D reconstruction setups typically rely on high-precision and costly hardware, such as 3D scanners like Artec Eva [33], to accurately capture both the topological and visual features of objects and scenes. The quality of the reconstruction is also highly dependent on the acquisition environment. For instance, key parameters, including camera positioning, lighting conditions, and surface reflectivity, need to be known in advance and consistently maintained to ensure the fidelity and reliability of the reconstruction process [34,35,36,37].

Nowadays, obtaining a representation of real or imaginary objects is expensive and time-consuming. Manual 3D modeling and designing are still the best choice for a lot of applications, with the use of Computer-Aided Design (CAD) or Digital Content Creation (DCC) software tools [38], however automatic reconstruction is rapidly gaining importance [7,39].

New technologies and hardware innovation allow a faster and more accurate reconstruction of 3D objects starting from simple sensory information such as images, point clouds and laser reflection signals [39,40,41]. In the last decade, Neural Implicit representations [42,43] unlocked the use of casual videos to reconstruct complex scenes with high visual accuracy by optimizing the scene as continuous volume rendering functions. In this scenario, 3D Gaussian Splatting (3DGS) [44] has emerged as a novel scene representation method, in which a set of 3D Gaussian primitives is optimized to adjust their scale, orientation, and color, to accurately reconstruct a scene based on available visual data [45,46]. This approach enables highly realistic scene rendering and supports real-time exploration and rapid novel view synthesis by efficiently processing millions of Gaussians.

This paper investigates how the initialization of the optimization process affects the quality of 3DGS reconstruction, an aspect that is often overlooked in the literature on Gaussian Splatting. We show that initialization has a measurable impact on the final representation, particularly in the case of isolated object reconstruction. Specifically, it improves quantitative performance in the early stages of optimization and qualitatively mitigates artifacts in the final reconstruction.

We propose Depth-initialized Gaussian Splatting (DiGS), a pipeline that integrates a depth-enhanced initialization method, leveraging depth data from a stereo camera, together with a segmentation algorithm for single-object reconstruction, differing from the standard Gaussian Splatting pipeline. We demonstrate that the proposed depth-based initialization produces a denser and more accurate point cloud of the target object while effectively filtering out background points. This results in improved reconstruction fidelity in the early stages of optimization, reducing artifacts caused by misclassified background regions and promoting a more uniform distribution of Gaussians in textureless areas. Notably, the proposed approach introduces a minimal overhead, does not require GPU acceleration, and remains low-cost, as it operates with affordable handheld stereo cameras.

In the experimental section, we compare the depth-enhanced initialization against two baselines: the standard 3DGS initialization [44,47] and a random initialization. Reconstruction quality is evaluated using standard quantitative metrics (i.e., PSNR, SSIM, and LPIPS) as well as subjective assessments obtained through a user study involving 20 participants. Experiments are conducted on a synthetic dataset derived from a subset of ShapeNet [48], including objects with varying geometries and categories. Additionally, we evaluate the method on a real-world dataset of luxury fashion garments to assess its ability to reconstruct fine-grained and highly structured objects.

Our contributions can be summarized as follows:

- We provide a systematic analysis of the impact of initialization in 3D Gaussian Splatting, demonstrating its critical role in both early-stage optimization and final reconstruction quality.
- We propose DiGS, a depth-based initialization pipeline that leverages RGB-D data to generate dense, scale-consistent, and noise-filtered point clouds, improving the quality of the initial Gaussian distribution.
- We introduce an initialization strategy that operates independently of the optimization process, enabling seamless integration with existing 3DGS pipelines without additional training overhead.
- We design a pipeline tailored for single-object reconstruction, combining segmentation and depth filtering to reduce background artifacts and improve geometric consistency.
- We conduct extensive experiments on both synthetic and real-world datasets, including a user study, showing that our method significantly improves reconstruction quality in early iterations while maintaining comparable final performance.

## 2. Related Work

The task of reconstructing 3D digital objects has received significant attention, with numerous methods proposed in the literature. In this section, we provide a brief overview of the most effective techniques, focusing on approaches that leverage depth information, either acquired through dedicated hardware or estimated from RGB data.

**Traditional Methods**. Traditional methods do not rely on Artificial Intelligence (AI)-based paradigms for 3D reconstruction; instead, they attempt to reconstruct objects using calibrated camera setups. State-of-the-art industrial methods use calibrated depth sensing cameras from multiple views to reconstruct the surface point cloud of the object and merge the different views in a coherent point cloud. More precisely, geometric information is usually obtained using laser-based sensors [49,50], structured light sensors [33,51,52] or stereo camera setups [53], while standard RGB cameras provide visual information. Alternatively, there exist methods capable of estimating the 3D structure directly from RGB images. Multi-view Stereo (MVS) [37] optimization solves the reprojection of points in the reference frame using both RGB images and known camera poses to generate depth maps and dense point clouds. The camera poses are usually obtained through Structure-from-motion (SfM) [47] which reconstructs a sparse point cloud and estimates the transformations between unstructured views by matching keypoints. However, both approaches require high image overlap and many views, and they struggle with low-texture surfaces and occlusions. Moreover, they do not model lighting effects, resulting in reconstructions that lack realism and photometric consistency when rendered from novel viewpoints [54,55].

**Neural implicit models.** Neural Radiance Field (NeRF) approaches [43] use the different views with known positions obtained from SfM to globally optimize a network that represent the volumetric field of the scene. They require long training time and rendering time but achieve high quality visual reconstruction [43,56]. However, the long training time and the slow rendering frame-rate made them unfeasible for most kinds of applications, where the representations are required to be interactable in real-time. Several techniques have been used to speed up the optimization and rendering computations leveraging smart discretization models to approximate the continuous field [57,58,59]. Depth maps of real scenes have been used to regularize the optimization process and obtain faster and better quality reconstruction [60,61]. Moreover, the absence of an explicit representation further limits the usage of NeRF in scenarios where geometric manipulation or integration into standard graphics pipelines is needed [62,63,64]. An alternative approach like Gaussian Splatting provides an explicit and lightweight scene representation, aiming to preserve the reconstruction quality of NeRF while enabling real-time rendering and easier integration into standard graphics workflows.

**Gaussian Splatting models.** 3DGS [44] has emerged as a highly efficient scene representation technique, modeling the environment as a collection of Gaussian primitives with optimizable parameters. This approach enables rapid optimization and real-time rendering, offering superior usability and visual fidelity compared to NeRF-based methods [46]. Recent works have further improved its practical deployment by introducing optimized implementations and training strategies [65]. Despite its advantages, 3DGS remains challenging to integrate into standard applications due to its reliance on high-performance GPUs and custom rasterization pipelines [46,66]. Our approach leverages depth-based initialization to ease GPU workload and reduces the number of iterations required during optimization.

To enhance geometric accuracy and practical usability, recent works have explored various regularization strategies during optimization. For instance, SuGaR [66] incorporates surface normals of neighboring Gaussians to promote local smoothness, while RaDe-GS [67] introduces a rasterization-based loss using both normal and depth maps. DN-Splatter [68] leverages real sensor depth data to guide the optimization process, combining measured depth and estimated normals in the loss function. Similarly, the method proposed in [69] utilizes monocular depth predictions from pretrained networks as geometric priors, although such depth maps, being computed frame by frame, often lack temporal consistency across views and introduce multi-view inconsistencies. More recent works have extended Gaussian Splatting beyond geometric reconstruction toward semantic and multimodal understanding. For instance, in [70], the authors integrate visual-language features into a Gaussian representation, enabling 3D reasoning and question answering directly from learned Gaussian fields.

However, the majority of prior works aim to improve final reconstruction quality through regularization during optimization, and few explicitly address the role of initialization. In particular, DN-Splatter adds computational cost during the splatting optimization cycle due to the additional depth and normal rasterization steps. The work does not address a different initialization method nor investigate the effect of the method on different training steps, setting the reached convergence to be reached at 30k iterations (standard Gaussian Splatting approximations). In our work, we focus on the initialization, ensuring flexibility with different optimization techniques and giving insights into the technique per se, analyzing the effect at different steps. The technique presented in [69] adds a depth rasterization step during the optimization, although the time cost for the additional computation is not reported. Additionally, the work does not report the generalization effects of the technique by comparing it with the standard multi-shot image paradigm, which, in general, scores higher in reconstruction quality.

To the best of our knowledge, only RAIN-GS [71] highlights the impact of initialization on the final results, proposing a strategy to relax the dependence on accurate camera poses and initial point clouds. However, it does so by introducing additional computational complexity during training, rather than improving the initial conditions themselves as we do in our work. Moreover, existing methods [66,67] do not explicitly target single-object reconstruction. Our experiments on real-world data reveal that these models often yield imprecise or noisy reconstructions when objects are isolated, especially in regions that remain unobserved across input views and thus are not updated during optimization. This leads to persistent noise and unreconstructed areas. Additionally, traditional SfM-based initialization [47], while effective in many scenarios, tends to rely on background features with high texture content, potentially introducing unwanted structures into the optimization—even when masking is applied. This study suggests that improving the quality of the initial point cloud, especially in the context of single-object reconstruction, can significantly enhance the final output. Our approach is based on using real, temporally consistent depth data to guide the generation of accurate surface normals, resulting in cleaner, more stable reconstructions. This hypothesis is supported by our experimental results, which demonstrate notable improvements in both geometric fidelity and visual coherence.

In Table 1, we summarize the discussed NeRF- and 3DGS-based methods, outlining their key features in comparison with our approach.

## 3. Proposed Method

**Figure 1:**
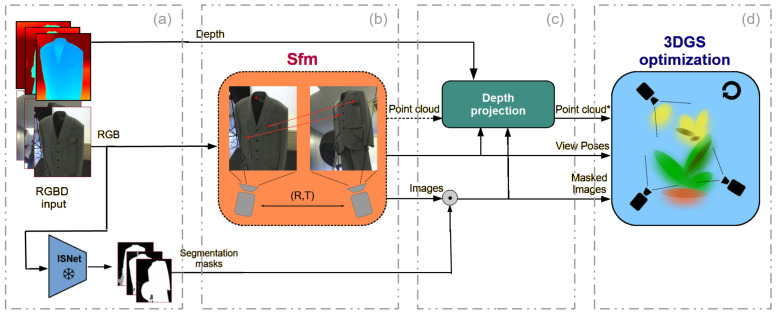
Overview of the proposed DiGS. The diagram illustrates the full pipeline, including the image preprocessing stage (**a**), camera pose estimation stage (**b**), initialization stage (**c**), and the subsequent 3DGS optimization stage (**d**). The point cloud obtained from standard SfM (**b**) is enhanced by projecting depth information (**c**) into the point cloud, creating a denser and more accurate starting point for the next stage, called Point cloud* in the figure. A pretrained segmentation mask model is used to separate the isolated object from the foreground, successfully reconstructing the object without the scene (**a**). Masks are applied to the images with the standard masking operator ⊙.

**Figure 2:**
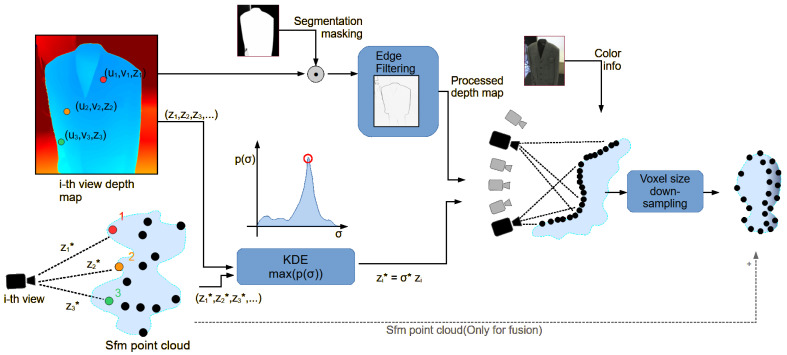
Diagram of the depth-enhanced initialization algorithm. The process begins with the estimation of a real-world scale factor, followed by the application of segmentation masks and edge-based filtering to each depth map to remove unwanted and noisy data from the acquisition process. Subsequently, the filtered depth maps are projected into a common reference frame, yielding a dense surface reconstruction. A voxel-based downsampling stage is then applied to regulate the point cloud density and reduce overrepresented regions. In the case of the Fusion experiment, the original SfM-derived point cloud is merged with the resulting geometry through a union operation.

The standard 3DGS framework reconstructs a representation of the entire captured scene from a monocular video by optimizing the parameters of a set of 3D Gaussian functions initialized from a sparse SfM point cloud [44]. The proposed *Depth-initialized Gaussian Splatting* (DiGS) pipeline (see Figure 1) extends this approach. Our method incorporates an Image Preprocessing stage where object segmentation extracts the foreground object, the Camera Pose Estimation stage using the SfM, the Initialization stage which provides custom point cloud initializations (see Figure 2), and finally the 3DGS Optimization stage.

### 3.1. Image Preprocessing

Foreground–background separation plays a crucial role in single-object reconstruction, as improper masking can introduce background artifacts or eliminate relevant surface geometry. Most automatic segmentation methods introduce some degree of inaccuracy, resulting in residual outliers or overcropped geometries that degrade reconstruction quality [72].

To balance pose estimation robustness and foreground fidelity, we propose applying segmentation after the SfM stage. This allows background features to contribute to robust keypoint matching and pose estimation. Then, the 3DGS optimization accommodates segmentation masks by ignoring gradients in masked-out regions (typically rendered as black or white). Formally, for a given input image $I_{i}\in R^{W\times H\times 3}$, a segmentation mask $S_{i}\in R^{W\times H}$ is computed via a segmentation function *G* with a set of parameters θ:

$$S_{i}=G\left(I_{i},\theta \right).$$

The masked image ${\bar{I}}_{i}$ is then defined at pixel coordinates $U\in R^{2}$ as:

$${\bar{I}}_{i}\left(U\right)=\left\{\begin{array}{cc} \mathrm{white}\;\mathrm{or}\;\mathrm{black} & \mathrm{if}\;S_{i}\left(U\right)<\tau \\ I_{i}\left(U\right) & \mathrm{otherwise}, \end{array}\right.$$

where τ is a threshold parameter used to binarize the mask output (typically in [0,1]).

### 3.2. Camera Pose Estimation

The Camera Poses are estimated using the Structure-from-Motion paradigm [47] applied to the RGB input. SfM is a multi-view geometry technique that jointly estimates camera intrinsics, poses, and a sparse 3D point cloud by triangulating matched keypoints across a sequence of uncalibrated images.

Given a collection of *N* images $I=\{I_{1},I_{2},\ldots ,I_{N}\}$, the SfM process recovers a set of 3D points $P=\left\{X_{1},\ldots ,X_{M}\right\}\subset R^{3}$ and the corresponding camera matrices $C={\left\{A_{i}M_{i}\right\}}_{i=1}^{N}$ such that each image projection $U_{ij}\in R^{2}$ of point $X_{j}$ in image $I_{i}$ satisfies the projection model:

(1) $$U_{ij}∼A_{i}M_{i}X_{j},$$

where ∼ denotes equality up to scale, $M_{i}\in R^{3\times 4}$ is the extrinsic matrix, and $A_{i}\in R^{3\times 3}$ is the intrinsic matrix of camera *i*.

The pipeline consists of:

1. **Feature detection and matching:** detecting and matching local descriptors, e.g., SIFT and ORB [73], to establish correspondences across image pairs.
2. **Initial reconstruction:** estimating the relative pose of an initial image pair and triangulating an initial sparse point cloud using robust estimation, e.g., RANSAC [74].
3. **Incremental registration:** adding new views via Perspective-n-Point (PnP) techniques [75] and expanding the 3D point cloud.
4. **Bundle adjustment:** refining all camera parameters and 3D point locations by minimizing the reprojection error:
  (2) $$A_{i}^{*},M_{i}^{*},X_{j}^{*}=\underset{A_{i},M_{i},X_{j}}{\arg\min}\sum_{i,j}{∥U_{ij}-\pi \left(A_{i}M_{i}X_{j}\right)∥}^{2},$$
  where π(·) denotes projection to image coordinates. For readability, the solutions of the optimization problem (2) are referred to as $A_{i},M_{i},X_{j}$.

In the following, we refer to the set of 3D points obtained from the SfM initialization as $P_{\mathrm{SfM}}$. SfM yields a sparse 3D representation suitable for initialization but lacks metric scale unless camera intrinsics or external measurements are provided. The resulting point cloud is populated by points that have, per-view, the highest information in the surroundings. These points are concentrated around corners and highly textured areas while flat, even regions are almost empty [76].

### 3.3. Initialization

In this section we describe two initialization strategies for generating point clouds prior to optimization: a depth-based approach that leverages sensor data and a fused method combining depth and SfM estimates.

#### 3.3.1. Depth-Based Initialization

To incorporate real-world-scale and higher point density, we integrate depth measurements captured during acquisition. As the SfM reconstruction provides only a scale-ambiguous geometry, we compute a global scale factor $\sigma^{*}$ by aligning the SfM-derived depths with those from depth sensors. The SfM point cloud is referred to as $P_{\mathrm{SfM}}$ in the following.

For each 3D point $X_{j}=\left[x_{j}\;y_{j}\;z_{j}\right]\in P_{\mathrm{SfM}}$, and for each view $I_{i}$ in which $X_{j}$ is visible at coordinate $U_{ij}=\left[u_{ij}\;v_{ij}\right]$, we compute the projected depth $d_{U_{ij}}^{*}$ using the camera pose transformation from the world reference frame (WRF) to the camera reference frame (CRF):

$$\mathrm{with}\;{\left[\begin{array}{c} {\tilde{x}}_{j}\\ {\tilde{y}}_{j}\\ {\tilde{z}}_{j}\\ 1 \end{array}\right]}_{CRF}=M_{i}{\left[\begin{array}{c} x_{j}\\ y_{j}\\ z_{j}\\ 1 \end{array}\right]}_{WRF}\;\Rightarrow \;d_{U_{ij}}^{*}={\tilde{z}}_{j}$$

Let $d_{U_{ij}}$ denote the depth measured by the camera at the *i*-th view at pixel position $U_{ij}$. Then, the local scale is:

$$\sigma_{ij}=\frac{d_{U_{ij}}^{*}}{d_{U_{ij}}}.$$

In theory, only one correspondence point is necessary, but the input data $d_{U_{ij}}$ is commonly noisy. The noise from RGB-D cameras often presents random noise, missing values, and outliers. To overcome this problem, we decided to use Kernel Density Estimation (KDE) over the full set of valid correspondences across the different images. The desired global $\sigma^{*}$ is obtained by fitting the Kernel Density Estimation (KDE) [77] over the set of all valid $\sigma_{ij}$ estimates and selecting the most probable value:

(3) $$\sigma^{*}=\arg\max_{\sigma }\;\mathrm{KDE}\;\left({\left\{\sigma_{ij}|X_{j}\;\mathrm{visible}\;\mathrm{in}\;I_{i}\right\}}_{i=1,\ldots ,N;\;j=1,\ldots ,M}\right).$$

This strategy ensures robustness to noise and incomplete depth measurements. The set of local scale estimates $\left\{\sigma_{ij}\right\}$ may contain outliers due to sensor noise or occlusions; however, selecting the highest-density peak of their distribution across all the views mitigates their impact. Random noise—often modeled as a Gaussian distribution—is mitigated by selecting the most probable value, corresponding to the zero mean of the Gaussian noise function. The set of values over which the KDE is applied is large on the order of $10^{6}$ across multiple shots, further reducing local frame noise. Furthermore, unreliable depth samples are pre-filtered using segmentation masks, edge-based criteria, and missing value removal, enhancing stability in real-world scenarios.

Using $\sigma^{*}$, we back-project each valid pixel at position *U* of depth map $D_{i}$ into world coordinates, obtaining the new point cloud $P_{i}^{*}=\left\{X_{i1}^{*},\ldots ,X_{iK}^{*}\right\}$:

(4) $$\left[\begin{array}{c} x_{ik}^{*}\\ y_{ik}^{*}\\ z_{ik}^{*}\\ 1 \end{array}\right]=M_{i}^{-1}A_{i}^{-1}\left[\begin{array}{c} u_{ik}\\ v_{ik}\\ \sigma^{*}d_{U_{ik}} \end{array}\right]$$

The large number of images from the different views is over-representative of the object surface; thus, from each view, we focus on the most reliable part of the depth map. We apply an edge-based filter using a Laplacian kernel on the depth map to remove unreliable measurements at object boundaries, setting $\tau_{\mathrm{edge}}=\mu \left(E_{i}\right)+\frac{1}{10}\sigma \left(E_{i}\right)$, where $E_{i}$ is the results of the Laplacian kernel applied to image $I_{i}$. A combination of a segmentation mask and two-centroid k-means clustering ensures that only foreground information filters in the final point cloud, without background outliers. $S_{i}$ is the segmentation mask associated with image $I_{i}$ obtained before. $C_{\mathrm{foreground}}$ is the set of pixels belonging to the foreground cluster obtained from the k-means algorithm. The selected subsample to be reprojected is only the pixel points that fulfill all the aforementioned conditions:

(5) $$U_{ik}\;\;\mathrm{such}\;\mathrm{that}\;\;S_{i}\left(U_{ik}\right)>\tau \;\wedge \;\left|E_{i}\left(U_{ik}\right)\right|<\tau_{\mathrm{edge}}\;\wedge \;U_{ik}\in C_{\mathrm{foreground}}.$$

To reduce over-representation of the surface, each projected point inherits its color from the RGB image, and per-view point clouds $P_{i}^{*}$ are merged and downsampled via a voxel grid filter *T* with voxel size γ:

(6) $$P^{*}=T_{\gamma }\left(\overset{N}{\underset{i=1}{⋃}}P_{i}^{*}\right).$$

Two downsampling levels are considered, namely γ=0.1 (low density) and γ=0.04 (high density).

#### 3.3.2. Fusion Initialization

To leverage the complementary strengths of depth-based and SfM representations—namely, dense surface coverage and high-frequency texture detail, respectively—we construct a fused point cloud by combining the depth-initialized point cloud with SfM-derived information, denoted as $P_{\mathrm{fusion}}^{*}$.

$P_{\mathrm{fusion}}^{*}$ is obtained as the union of the downsampled depth-based point cloud $P^{*}$ and the filtered subset of SfM points $P_{\mathrm{BB}}$, where $P_{\mathrm{BB}}$ includes only points that are not masked in any of the views in which they appear. No explicit confidence weighting is introduced between the two sources; instead, both contribute equally via the set-union operation, defined as follows:

$$P_{\mathrm{fusion}}^{*}=P_{\mathrm{BB}}\cup P^{*}.$$

The filtered subset of the SfM point cloud is used to avoid infiltration of outliers points from the background in the final point cloud. The filter is the following:

$$P_{\mathrm{BB}}\subset P_{\mathrm{SfM}},\;P_{\mathrm{BB}}=\left\{X_{j}\in P_{\mathrm{SfM}}\;|\;S_{i}\left(U_{ij}\right)>\tau \wedge \;X_{j}\in BB_{P^{*}}\right\},$$

where $BB_{P^{*}}$ denotes the smallest bounding box enclosing all points in $P^{*}$; and $S_{i}\left(U_{ij}\right)$ is the mask value of the re-projected pixel coordinates $U_{ij}$ for image $I_{i}$ associated with $X_{j}$.

### 3.4. 3DGS Optimization

The final point clouds $P^{*}$ and $P_{\mathrm{fusion}}^{*}$ serve as initializations for the 3D Gaussian Splatting (3DGS) pipeline [43]. Each point initializes the center μ and the harmonic color coefficients of a 3D Gaussian, defined as:

$$G\left(x\right)=e^{-\frac{1}{2}{\left(x\right)}^{⊤}\Sigma \left(x\right)}$$

where Σ is the covariance matrix and $\mu \in R^{3}$ denotes the Gaussian center.

In the following, we denote the set of Gaussian centers as $P_{\mu }\subset R^{3}$, which corresponds to the geometric structure derived from the final point cloud.

The optimization follows the original 3DGS formulation and aims to minimize a combined photometric loss:

$$L=\left(1-\lambda \right)L_{1}+\lambda L_{D-\mathrm{SSIM}},$$

where $L_{1}$ is the pixel-wise absolute difference (L1 loss) between rendered and ground-truth images, and $L_{\text{D-SSIM}}$ is a differentiable structural similarity index (D-SSIM) that measures perceptual quality. Both losses are computed across all training views to guide the optimization of Gaussian parameters.

## 4. Experiments and Evaluation

In this section, we present the experiments conducted to validate our approach against alternative initialization strategies. We provide details on the experimental setup, quantitative and qualitative evaluations, and user study results to assess reconstruction quality under various conditions.

### 4.1. Experimental Setup

Experiments were carried out on two datasets: a subset of the ShapeNet [48] repository consisting of the 50 models with the highest vertex count and a custom real-world dataset comprising 10 fashion garments from the Italian brand Moschino, referred to as Moschino in the following.

The ShapeNet dataset was chosen to ensure complexity comparable to real objects, using vertex count as a proxy for geometric richness. To generate RGB-D data, each model was imported into Blender 4.0 [78], and a custom script was employed to simulate a camera moving along a noisy trajectory around the object, mimicking realistic data acquisition. To aid the SfM process in estimating accurate camera poses and to mitigate synthetic-to-real domain gaps, each object was enclosed in a textured sphere derived from a real 360° image, thus providing a photorealistic background.

The Moschino dataset was acquired using a ZED 2 stereo camera [79], leveraging its built-in hardware-accelerated depth refinement to record RGB-D videos. Each video sequence describes three circular trajectories at different elevations around the object. The camera was handheld, with real-time visual feedback provided to help maintain object centering. Acquisitions were performed in a semi-controlled indoor environment with non-professional lighting and the presence of background clutter, to maintain generalization with “in-the-wild” videos.

From each video in the datasets considered, 200 uniformly spaced frames were extracted and used as input images, along with their corresponding depth maps. Input images were initially captured in 2K resolution for preprocessing and subsequently downsampled to 1.6K resolution for optimization, consistent with the standard 3DGS pipeline. The segmentation masks were obtained using the pretrained *isnet-general-use* model [80], chosen for its strong generalization capabilities in foreground extraction and its ability to preserve complex geometric details.

Three depth-enhanced initialization strategies were evaluated: two differing in projected point cloud density using voxel sizes γ=0.04 (high density) and γ=0.1 (low density), referred to as the **Highdepth** and **Depth**, respectively. The third strategy, named **Fusion**, incorporates the SfM-derived point cloud into the depth-based representation. These initializations were compared against a baseline 3DGS setup using only segmentation masks and a randomly initialized pipeline which are referred to as **Default** and **Random**, respectively.

All experiments were conducted on a desktop workstation equipped with a 12-core AMD Ryzen 9 processor, 64 GB of RAM, and a 24 GB GPU. For comparative purposes, the pipeline was executed independently for each initialization method on the same object. Intermediate results were saved and reloaded at various pipeline stages; however, the time required for these I/O operations was excluded from the reported computation times.

We report the hyperparameters used in all experiments. All values are fixed across datasets and were empirically selected and kept constant throughout all experiments. The complete set of hyperparameters is summarized below:

- Mask threshold is set as τ=20.
- Laplacian kernel size equals 5.
- The parameter $\tau_{\mathrm{edge}}$ is set to 10% of the standard deviation of image gradients.
- KDE scale factor precision equals $10^{-3}$.
- The k-means weights used for centroid assignment equal $w_{\mathrm{foreground}}=1.0$ and $w_{\mathrm{background}}=3.0$.

### 4.2. Evaluation Metrics

The objective metrics are computed between the rendered views of the reconstructed 3D models and the corresponding views in the dataset at various iterations of the DiGS optimization process. Specifically, in the following we report the mean values averaged over both datasets, namely ShapeNet and Moschino. However, as highlighted in the recent literature, PSNR alone is not always indicative of perceptual quality. Therefore, we also include LPIPS and SSIM as complementary evaluation metrics [81].

To better evaluate reconstruction quality from a perceptual perspective, beyond numerical metrics, a user study with 20 partecipants was conducted to assess human preference among the different initialization strategies. Each participant viewed side-by-side videos showing the reconstructions of the same object using the four initialization approaches under comparison. The videos were arranged in a 2 × 2 grid, with all models rotating synchronously to reveal all visible surfaces. Two reference images, common to all participants and selected from the input set, were displayed alongside the video. Each video showed the objects at a fixed optimization iteration. To prevent bias, the spatial position of each method in the grid was randomized according to a Latin square design and kept hidden from the participants. The test explanation didn’t include any information about the technique used or the expected results. Finally, participants were recruited on a voluntary basis through internal university mailing lists and word of mouth.

### 4.3. Results and Discussion

In this section, we compare different initializations through image-to-image comparisons across multiple viewpoints. To analyze their influence throughout the optimization process, the results were collected at several iteration steps (specifically at 100, 300, 500, 1000, 3000, 7000, and 12,000 iterations) for both datasets. In Table 2 we report the mean values of the considered objective metrics computed for all the elements in the ShapeNet and Moschino datasets. In Table 3 we report the mean performance across the different datasets to appreciate the differences between the real and the synthetic data.

These results show that the choice of point cloud initialization significantly affects the optimization outcomes. Among all tested strategies, the Highdepth initialization consistently achieves the best performance across all metrics and iterations, showing higher PSNR and SSIM, and lower LPIPS values. The Fusion strategy also performs as good as Highdepth. The Default and Depth initializations offer reasonable results but tend to underperform compared to Highdepth and Fusion, especially in early iterations. These outcomes suggest that leveraging more accurate and denser depth information provides a strong prior for reconstruction quality. Lastly, the Random initialization performs the worst in early stages and only begins to approach acceptable quality after many iterations. This reinforces the notion that poor initialization may slow down convergence and limit performance. However, as the number of iterations increases, the performance gap in terms of PSNR, SSIM and LPIPS among the different initialization strategies narrows. Indeed, beyond 7000 iterations, most methods achieve comparable metric values. This indicates that while the optimization process is able to refine all initializations over time, stronger priors such as Highdepth enable reaching high-quality results with fewer iterations. Therefore, this initial test highlights that the choice of initialization is particularly impactful in the early phase and can be crucial for obtaining reliable outcomes more efficiently.

**Figure 3:**
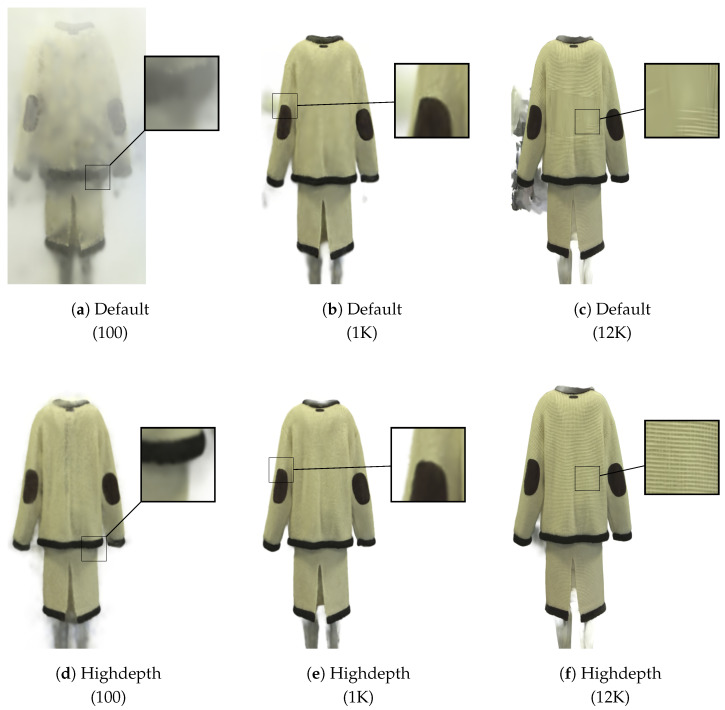
Visual comparisons between Default and Highdepth initializations at different iterations during the optimization process. The iterations are indicated in parentheses. From the comparison between the Default initialization (**a**–**c**) and the Highdepth initialization (**d**–**f**), a better fine-grained texture reconstruction can be observed. Comparing (**a**,**d**), the effect of the improved performance in the early iterations is evident. In (**c**), the knitting texture on the back of the garment is not correctly reconstructed. In (**b**,**c**), artifacts are visible near the left sleeve, which are significantly reduced in (**e**,**f**).

A visual comparison of the reconstruction for an element within the Moschino dataset is shown in Figure 3. The Highdepth initialization is compared against the Default at various stages of the optimization process (iterations 100, 1000, and 12,000). It is immediately evident that in the early iterations, Highdepth leads to sharper object boundaries and improved detail fidelity. Interestingly, this figure also reveals an aspect not fully captured by the quantitative metrics reported in Table 2 at 12,000 iterations: Highdepth remains more effective at reconstructing fine-grained textures, particularly in regions with complex patterns (see close-ups in Figure 3c,f). This qualitative advantage highlights the benefits of depth-based initializations, especially when dealing with intricate appearance details. We remark that these observations generalize well across all datasets.

**Figure 4:**
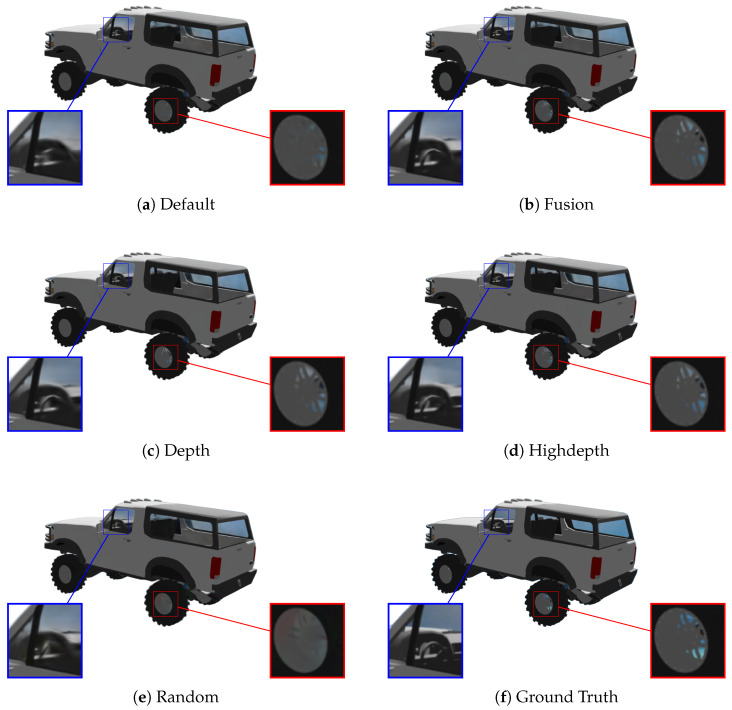
Visual comparison at 12K iterations for different initialization strategies (Default, Fusion, Depth, Highdepth, and Random) with the Ground Truth image (**f**). Depth-based reconstruction methods (**b**–**d**) better reconstruct the details and thin structures of the wheel rims and the steering wheel compared to the random (**e**) and default initialization (**a**). Depth-based initialization provides accurate seeding for the Gaussians, enabling the correct reconstruction of otherwise underrepresented regions.

In Figure 4, we show the final outputs of different initialization strategies on a sample object from the ShapeNet dataset. The depth-based initializations (Depth, Highdepth, and Fusion) produce reconstructions that more accurately preserve fine details and complex structures, particularly in highly cluttered regions, when compared to the Ground Truth. This confirms the advantage of leveraging geometric priors even at later stages of optimization.

**Figure 5:**
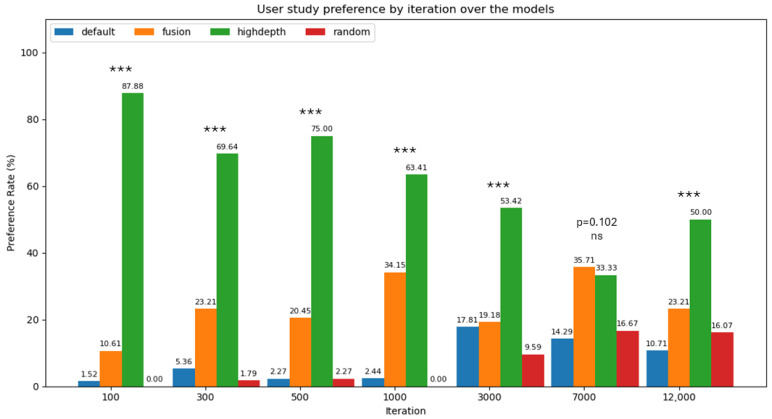
Bar plot highlighting the preference scores from the user study comparing reconstruction quality across four different initialization methods: Default, Highdepth, Fusion, and Random. Preference is expressed as a rate (%) corresponding to the selection of the best reconstruction among the methods at the same iteration. The Highdepth initialization is the method that achieves the highest preference for most iterations. Not only in the early stages, but also in the later stages, the preference remains significant, in contrast with the quantitative results, where the differences among methods are almost negligible. Statistical significance was assessed using a chi-square test at each iteration: differences are significant in all cases (***, p<0.001) except at 7000 iterations (ns, p=0.102), where user preferences are not significantly different.

To further support the qualitative observations reported in Figure 3 and Figure 4, in Figure 5 we present the results of a user study involving 20 participants, who were asked to express their visual preference among models reconstructed using different initialization methods, Default, Highdepth, Random, and Fusion, across several optimization iterations on the ShapeNet dataset. Furthermore, Figure 5 reports the statistical differences assessed using a chi-square test. The results clearly indicate that the Highdepth initialization consistently outperformed the others in terms of user preference, especially in the early training stages (e.g., iterations 100 to 1000), where it achieved exceptionally high scores, peaking at 87.88% at iteration 100. Notably, Fusion emerged as a competitive alternative at later stages, briefly surpassing Highdepth at iteration 7000, suggesting that its advantages may emerge more gradually with training. In contrast, both Default and Random methods consistently received low preference rates across all iterations, highlighting the effectiveness of Highdepth (especially early) and the potential of Fusion in later stages.

**Figure 6:**
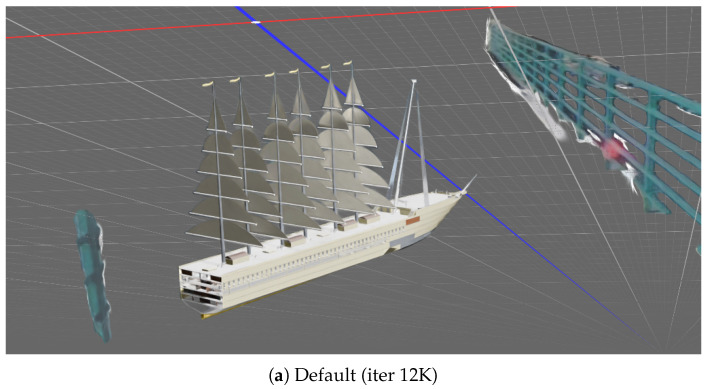

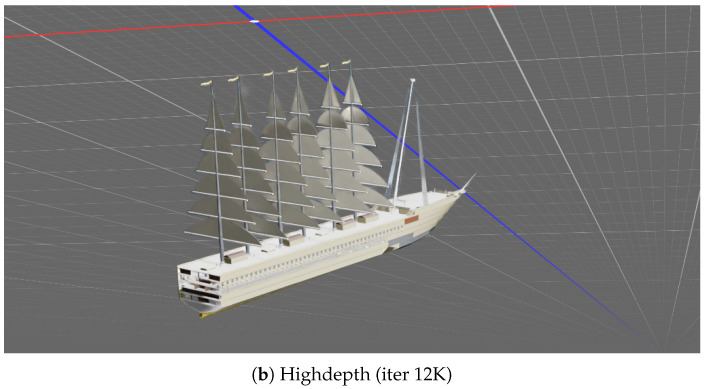
Comparison example of reconstructions for the Default and Highdepth initializations. Some objects that are not correctly masked may propagate into the final reconstruction, for example the cyan artifact on the right side of image (**a**). The filtering mechanism implemented in the depth-based initializations can successfully mitigate these effects, which are not present in image (**b**). The colored lines correspond to a background reference grid used for visualization.

Moreover, our approach demonstrates robustness to segmentation errors in the input images. As shown in Figure 6, the proposed depth-based pipeline effectively filters out background and outlier points, preventing the persistence of erroneous Gaussians in the final result. In contrast, the baseline method is penalized by incorrect segmentations, as background regions incorrectly included in the ground truth contribute negatively to loss values.

**Figure 7:**
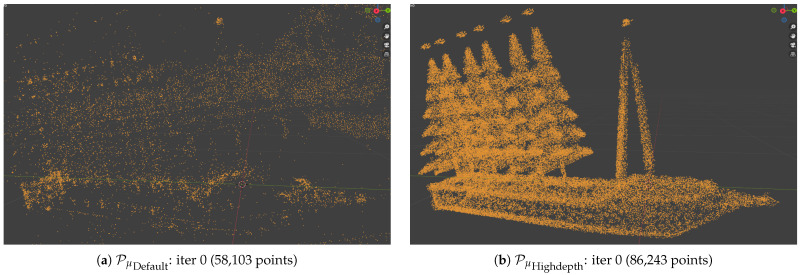

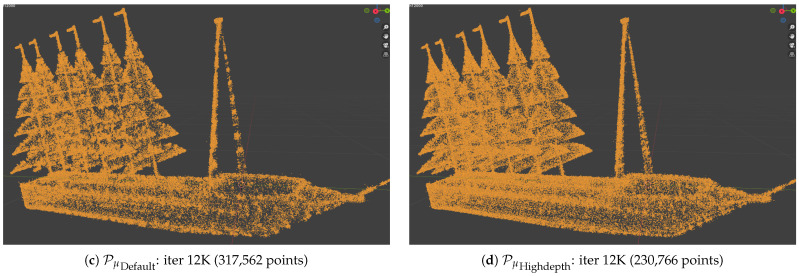
Visual comparison of the distribution of Gaussian centers at the initial and final iterations for Highdepth and Default. The SfM-based initial reconstruction focuses only on image-to-image correspondence points and relies heavily on background correspondences (**a**). Our methods generate a denser point cloud in the region of interest with comparable cardinality, completely removing unwanted background points (**b**). At the end of the reconstruction, Gaussian centers are more evenly distributed, enabling better reconstruction of thin structures with our method (**d**), while the optimization struggles with the default initial point cloud and exhibits a sparse, uneven distribution of Gaussians (**c**).

We now analyze the distributions $P_{\mu }$ of Gaussian centers for Default and Highdepth initializations. For an object within the ShapeNet dataset, in Figure 7 we show that the proposed Highdepth initialization results in a denser and more uniform coverage, including low-textured regions. Unlike the Default approach, which tends to concentrate Gaussians in high-textured areas, Highdepth more accurately captures the full topology of the object across all surfaces. The Highdepth initialization (see Figure 7b) achieves a more faithful approximation of the object’s surface compared to the Default (see Figure 7a). After full optimization using standard 3DGS (see Figure 7c), Gaussians cluster around object corners, whereas our proposal (see Figure 7d) produces a more uniform coverage, despite using fewer Gaussians overall. Such a distribution may be advantageous for downstream tasks like mesh extraction, although this was not deeply investigated in the present study. We remark that the Default approach uses the standard Adaptive Density Control (ADC) mechanism [44] which often fails to adequately populate under-sampled regions with sufficient Gaussians. In contrast, our Highdepth initialization provides a depth-based prior that guides the optimization towards more accurate reconstructions.

Finally, concerning the computational time for the whole process, we remark that the proposed method introduces a preprocessing step that increases the total reconstruction time, as reported in Table 4. However, this step introduces a minimal overhead of only 13 s, roughly equivalent to 500 optimization iterations, when executed using non-GPU-dependent code. On systems with lower computational capacity, where each optimization step is more time-consuming, the reduction in required iterations may lead to a gain in efficiency. Additionally, the acquisition of depth data was performed using an affordable, professional-grade stereo camera (ZED 2) priced at approximately 500 euros, comparable to mid-range smartphones. Thus, the inclusion of depth data does not significantly compromise the method’s accessibility.

## 5. Limitations and Future Work

Despite the promising results, we acknowledge several limitations of the present study that suggest directions for future research.

First, the evaluation focuses on comparing initialization strategies within the 3DGS framework, as the goal was to understand their impact on performance, an aspect not previously explored in the literature. Direct quantitative comparisons with alternative reconstruction paradigms and broader benchmarking across different frameworks are left for future work.

Second, the real-world dataset used in this work primarily consists of clothing objects, which are flexible and thin structures. While experiments on the ShapeNet dataset partially support generalization to rigid objects with diverse geometries and textures, further evaluation on real-world rigid objects, especially those with coarse textures, is left for future work.

Third, while the proposed method demonstrates strong performance, the contribution of each individual component in the pipeline (e.g., segmentation, edge filtering, KDE-based scale estimation, and voxel-grid downsampling) has not been explicitly isolated through a dedicated ablation study. We leave this investigation for future work.

Finally, future directions include integrating monocular depth estimation techniques to remove the dependency on stereo hardware, and exploring the utility of our initialization strategy in downstream tasks such as mesh extraction.

## 6. Conclusions

In this work, we presented a novel depth-enhanced initialization strategy for Gaussian Splatting, aimed at improving reconstruction quality and convergence speed in implicit 3D representations. By leveraging depth information acquired during RGB-D data capture, we proposed an initialization pipeline that integrates segmentation, scale estimation and multi-view depth map projection to generate a dense and structured point cloud. This initialization serves as a geometric prior, allowing for more accurate outcomes within the 3D Gaussian Splatting framework. The technique introduces a limited disadvantage in terms of time cost, lower than 1.5% of the total time cost.

Several experiments were conducted on both synthetic and real-world datasets, demonstrating that our method significantly improves reconstruction quality, particularly in early optimization stages. Quantitative metrics such as PSNR, SSIM and LPIPS confirmed consistent gains over standard and randomly-initialized baselines. Qualitative comparisons revealed sharper details, more accurate boundaries, and reduced clutter in reconstructions. The robustness of the proposed approach to segmentation errors was also highlighted, showing improved resilience to outliers and noise.

Additionally, we conducted a user study to evaluate perceptual preferences across different initialization strategies to better corroborate our findings. The results indicate a significant preference for the Highdepth, and in general depth-enhanced pipelines.

Importantly, the added computational cost introduced by our method remains minimal and acceptable, especially considering the significant reduction in required optimization iterations. Furthermore, the use of low-cost RGB-D hardware ensures that the approach remains accessible and scalable.

Overall, our method provides a practical and effective enhancement to the Gaussian Splatting pipeline, paving the way for more efficient and high-fidelity 3D reconstructions from unconstrained video input.

## Figures and Tables

**Table 1:** Comparison of state-of-the-art NeRF- and 3DGS-based methods for 3D reconstruction. The table highlights whether each method supports Few-shot input (FS), enables Mesh Extraction (ME), introduces an Initialization Strategy (IS), uses RGB-D data, supports Single-object Reconstruction (SOR), and achieves Real-time Rendering (RTR). A checkmark (✔) indicates the presence of a feature, while a cross (✘) indicates its absence.

	FS	ME	IS	RGB-D	SOR	RTR
**NeRF**						
Barron et al. [57]	✘	✘	✘	✘	✘	✘
Garbin et al. [59]	✘	✘	✘	✘	✘	✔
Li et al. [58]	✘	✔	✘	✘	✘	✘
Dent et al. [60]	✘	✘	✘	✔	✘	✘
Kwak et al. [61]	✔	✘	✘	✔	✘	✘
**3DGS**						
Guédon et al. [66]	✘	✔	✘	✘	✘	✔
Zhang et al. [67]	✘	✔	✘	✘	✘	✔
Turkulainen et al. [68]	✘	✔	✘	✔	✘	✔
Chung et al. [69]	✔	✘	✘	✔	✘	✔
Jung et al. [71]	✘	✘	✔	✘	✘	✔
***Ours***	✘	✘	✔	✔	✔	✔

**Table 2:** Dataset-averaged score comparison across different iterations for various point cloud initialization methods (Default, Depth, Highdepth, Fusion, and Random). The Highdepth initialization achieves the best performance among the proposed methods. Each depth-based initialization exhibits a significant performance boost in the early stages, while its impact diminishes in the later stages of optimization. Higher values are better for PSNR and SSIM (↓), while lower values are better for LPIPS (↑). Best results are highlighted in bold.

Iterations	100			500			1000		
	PSNR ↑	SSIM ↑	LPIPS ↓	PSNR ↑	SSIM ↑	LPIPS ↓	PSNR ↑	SSIM ↑	LPIPS ↓
Default	17.553	0.9027	0.1510	23.206	0.9300	0.0995	24.540	0.9419	0.0833
Depth	22.673	0.9275	0.1031	24.376	0.9400	0.0859	24.901	0.9450	0.0793
Highdepth	**23.629**	**0.9363**	**0.0909**	**24.922**	**0.9462**	**0.0777**	**25.235**	**0.9490**	**0.0740**
Fusion	22.885	0.9308	0.1023	24.711	0.9438	0.0810	25.130	0.9477	0.0753
Random	8.466	0.8019	0.3970	16.542	0.8977	0.1373	22.088	0.9229	0.1112
									
Iterations	3000			7000			12,000		
Default	25.697	0.9525	0.0696	26.675	0.9590	0.0611	27.393	0.9632	0.0564
Depth	25.848	0.9527	0.0688	26.764	0.9587	0.0608	27.519	0.9626	0.0563
Highdepth	**25.986**	**0.9544**	**0.0657**	**26.860**	**0.9602**	**0.0581**	**27.581**	**0.9636**	**0.0539**
Fusion	25.963	0.9540	0.0664	26.811	0.9597	0.0592	27.552	0.9633	0.0550
Random	25.207	0.9476	0.0767	26.563	0.9572	0.0636	27.392	0.9617	0.0581

**Table 3:** Mean score comparison for the Moschino and Synthetic datasets using the proposed Highdepth technique and the Default pipeline. Both datasets exhibit a similar pattern: an improvement in performance during the early iterations, followed by comparable convergence in the later stages. The Moschino dataset shows lower performance scores for both the baseline and the proposed method compared to the synthetic dataset, as real-world data are generally noisier and more challenging to reconstruct than synthetic data. Higher values are better for PSNR and SSIM (↓), while lower values are better for LPIPS (↑). Best results are highlighted in bold.

**Moschino Dataset**						
**Iterations**	**Highdepth**			**Default**		
	**PSNR ↑**	**SSIM ↑**	**LPIPS ↓**	**PSNR ↑**	**SSIM ↑**	**LPIPS ↓**
100	**19.243**	**0.8949**	**0.1798**	15.045	0.8648	0.2204
500	**20.011**	**0.9004**	**0.1607**	19.630	0.8941	0.1742
1000	**20.140**	**0.9023**	**0.1569**	20.087	0.8993	0.1656
3000	20.280	**0.9068**	**0.1488**	**20.372**	0.9054	0.1547
7000	20.498	**0.9141**	**0.1382**	**20.630**	0.9115	0.1455
12,000	20.685	**0.9193**	**0.1302**	**20.851**	0.9175	0.1379
**Synthetic Dataset**						
**Iterations**	**Highdepth**			**Default**		
	**PSNR ↑**	**SSIM ↑**	**LPIPS ↓**	**PSNR ↑**	**SSIM ↑**	**LPIPS ↓**
100	**24.944**	**0.9487**	**0.0642**	18.306	0.9140	0.1302
500	**26.395**	**0.9599**	**0.0528**	24.279	0.9408	0.0770
1000	**26.764**	**0.9630**	**0.0491**	25.8763	0.9547	0.0586
3000	**27.698**	**0.9687**	**0.0408**	27.295	0.9666	0.0441
7000	**28.769**	**0.9740**	**0.0341**	28.488	0.9733	0.0357
12,000	**29.650**	**0.9769**	**0.0311**	29.356	0.9769	0.0320

**Table 4:** Time cost for each initialization method and pipeline step, averaged over the dataset. The standard deviation over the dataset is also reported. The proposed depth-based methods add an initial computational overhead, which is low compared to the total reconstruction time and relies on non-GPU-dependent computation. The total reconstruction cost remains approximately consistent across the different methods, due to the fixed and computationally expensive SfM step.

		Default	Depth	Highdepth	Fusion	Random
**Processing** (Colmap & Masking)		759 ± 60 s	759 ± 60 s	759 ± 60 s	759 ± 60 s	759 ± 60 s
**Initialization**		−	12 ± 1 s	13 ± 1 s	13 ± 1 s	0 s
**Iteration**	100	4 ± 2 s	2 ± 1 s	2 ± 1 s	2 ± 1 s	10 ± 3 s
	300	10 ± 5 s	6 ± 3 s	8 ± 4 s	7 ± 4 s	26 ± 9 s
	500	16 ± 8 s	10 ± 5 s	13 ± 6 s	11 ± 6 s	37 ± 13 s
	1000	27 ± 11 s	20 ± 10 s	25 ± 13 s	22 ± 11 s	48 ± 17 s
	3000	67 ± 26 s	60 ± 28 s	70 ± 38 s	63 ± 29 s	86 ± 31 s
	7000	152 ± 54 s	144 ± 60 s	163 ± 79 s	149 ± 62 s	171 ± 60 s
	12,000	271 ± 96 s	262 ± 104 s	290 ± 134 s	268 ± 107 s	290 ± 103 s
**Total time**		1030 ± 121 s	1035 ± 127 s	1064 ± 154 s	1041 ± 131 s	1049 ± 126 s

## Data Availability

The subset of the ShapeNet dataset generated during and/or analyzed during the current study is available in the full form in the ShapeNet repository, https://huggingface.co/datasets/ShapeNet/ShapeNetCore (accessed on 4 February 2025). The fashion garment dataset generated during and/or analyzed during the current study is not publicly available due to the design being property of the brand, but it is available from the corresponding author on reasonable request.

## Abbreviations

The following abbreviations are used in this manuscript:

DiGS	Depth-initialized Gaussian Splatting
SfM	Structure from Motion
3DGS	3D Gaussian Splatting
SuGaR	Surface-aligned Gaussian Splatting
NeRF	Neural Radiance Field
KDE	Kernel Density Estimation
RGB	Red Green Blue
RGB-D	Red Green Blue Depth
SIFT	Scale-invariant transform function
ORB	Orientated FAST and Robust BRIEF
RANSAC	RANdom SAmple Consensus
PSNR	Peak Signal-to-Noise Ratio
LPIPS	Learned Perceptual Image Patch Similarity
SSIM	Structural Similarity Index Measure
RAM	Random Access Memory
GPU	Graphics Processing Unit
ADC	Adaptive Density Control
RaDe-GS	Rasterizing Depth in Gaussian Splatting
DN-Splatter	Depth and Normal Priors for Gaussian Splatting and Meshing
RAIN-GS	Relaxing Accurate Initialization Constraint for 3D Gaussian Splatting
MVS	Multi-view Stereo
CAD	Computer-aided Design
DCC	Digital Content Creation

## References

[1] Li K., Cui Y., Li W., Lv T., Yuan X., Li S., Ni W., Simsek M., Dressler F. (2022). When internet of things meets metaverse: Convergence of physical and cyber worlds. IEEE Internet Things J..

[2] Visconti R.M. (2022). From physical reality to the Metaverse: A Multilayer Network Valuation. J. Metaverse.

[3] Vallasciani G., Stacchio L., Cascarano P., Marfia G. (2024). CreAIXR: Fostering creativity with generative AI in XR environments. Proceedings of the 2024 IEEE International Conference on Metaverse Computing, Networking, and Applications (MetaCom).

[4] Hajahmadi S., Calvi I., Stacchiotti E., Cascarano P., Marfia G. (2024). Heritage elements and Artificial Intelligence as storytelling tools for virtual retail environments. Digit. Appl. Archaeol. Cult. Herit..

[5] Hajahmadi S., Stacchio L., Giacché A., Cascarano P., Marfia G. (2024). Investigating extended reality-powered digital twins for sequential instruction learning: The case of the rubik’s cube. Proceedings of the 2024 IEEE International Symposium on Mixed and Augmented Reality (ISMAR).

[6] Rodríguez-García B., Guillen-Sanz H., Checa D., Bustillo A. (2024). A systematic review of virtual 3D reconstructions of Cultural Heritage in immersive Virtual Reality. Multimed. Tools Appl..

[7] Phang J.T.S., Lim K.H., Chiong R.C.W. (2021). A review of three dimensional reconstruction techniques. Multimed. Tools Appl..

[8] Cascarano P., Meglioraldi J., Vallasciani G., Armandi V., Augello G., Carradori S., Hajahmadi S., Marfia G. (2025). A Comparative Analysis of 3D Modeling Methods for Integration into an Extended Reality Platform. Proceedings of the 2025 IEEE International Conference on Artificial Intelligence and Etended and Virtual Reality (AIxVR).

[9] Bruno F., Bruno S., De Sensi G., Luchi M.L., Mancuso S., Muzzupappa M. (2010). From 3D reconstruction to virtual reality: A complete methodology for digital archaeological exhibition. J. Cult. Herit..

[10] Collins J., Goel S., Deng K., Luthra A., Xu L., Gundogdu E., Zhang X., Vicente T.F.Y., Dideriksen T., Arora H. Abo: Dataset and benchmarks for real-world 3d object understanding. Proceedings of the IEEE/CVF Conference on Computer Vision and Pattern Recognition.

[11] Calli B., Singh A., Walsman A., Srinivasa S., Abbeel P., Dollar A.M. (2015). The ycb object and model set: Towards common benchmarks for manipulation research. Proceedings of the 2015 International Conference on Advanced Robotics (ICAR).

[12] Agnew W., Xie C., Walsman A., Murad O., Wang Y., Domingos P., Srinivasa S. (2021). Amodal 3d reconstruction for robotic manipulation via stability and connectivity. Proceedings of the Conference on Robot Learning.

[13] Iwase S., Irshad Z., Liu K., Guizilini V., Lee R., Ikeda T., Amma A., Nishiwaki K., Kitani K., Ambrus R. (2025). ZeroGrasp: Zero-Shot Shape Reconstruction Enabled Robotic Grasping. arXiv.

[14] Thrun S. (2002). Robotic mapping: A survey. Exploring Artificial Intelligence in the New Millennium.

[15] Wang T.W., Huang H.P., Zhao Y.L. (2025). Vision-Guided Autonomous Robot Navigation in Realistic 3D Dynamic Scenarios. Appl. Sci..

[16] Xu Z., Zhan X., Chen B., Xiu Y., Yang C., Shimada K. (2023). A real-time dynamic obstacle tracking and mapping system for UAV navigation and collision avoidance with an RGB-D camera. Proceedings of the 2023 IEEE International Conference on Robotics and Automation (ICRA).

[17] Gomes L., Bellon O.R.P., Silva L. (2014). 3D reconstruction methods for digital preservation of cultural heritage: A survey. Pattern Recognit. Lett..

[18] Kargas A., Karitsioti N., Loumos G. (2020). Reinventing museums in 21st century: Implementing augmented reality and virtual reality technologies alongside social Media’s logics. Virtual and Augmented Reality in Education, Art, and Museums.

[19] Kantaros A., Ganetsos T., Petrescu F.I.T. (2023). Three-dimensional printing and 3D scanning: Emerging technologies exhibiting high potential in the field of cultural heritage. Appl. Sci..

[20] Wachowiak M.J., Karas B.V. (2009). 3D scanning and replication for museum and cultural heritage applications. J. Am. Inst. Conserv..

[21] Weng J., Sun J. (2025). Green landscape 3D reconstruction and VR interactive art design experience using digital entertainment technology and entertainment gesture robots. Entertain. Comput..

[22] Zioulis N., Alexiadis D., Doumanoglou A., Louizis G., Apostolakis K., Zarpalas D., Daras P. (2016). 3D tele-immersion platform for interactive immersive experiences between remote users. Proceedings of the 2016 IEEE International Conference on Image Processing (ICIP).

[23] Li L., Carnell S., Harris K., Walters L., Reiners D., Cruz-Neira C. LIFT-A System to Create Mixed 360 Video and 3D Content for Live Immersive Virtual Field Trip. Proceedings of the 2023 ACM International Conference on Interactive Media Experiences.

[24] Richlan F., Weiß M., Kastner P., Braid J. (2023). Virtual training, real effects: A narrative review on sports performance enhancement through interventions in virtual reality. Front. Psychol..

[25] Huang X., Yin M., Xia Z., Xiao R. VirtualNexus: Enhancing 360-Degree Video AR/VR Collaboration with Environment Cutouts and Virtual Replicas. Proceedings of the 37th Annual ACM Symposium on User Interface Software and Technology.

[26] Wu Y., Yi A., Ma C., Chen L. (2023). Artificial intelligence for video game visualization, advancements, benefits and challenges. Math. Biosci. Eng..

[27] Huang Y. (2024). 3D special effects modelling based on computer graphics technology. Appl. Comput. Eng..

[28] Gui Z., Jha S., Delbos B., Moreau R., Chalard R., Lelevé A., Cheng I. (2024). Interactive Manipulation and Visualization of 3D Brain MRI for Surgical Training. Proceedings of the 2024 46th Annual International Conference of the IEEE Engineering in Medicine and Biology Society (EMBC).

[29] Pathak K., Saikia R., Das A., Das D., Islam M.A., Pramanik P., Parasar A., Borthakur P.P., Sarmah P., Saikia M. (2023). 3D printing in biomedicine: Advancing personalized care through additive manufacturing. Explor. Med..

[30] Clarke E. (2021). Virtual reality simulation—The future of orthopaedic training? A systematic review and narrative analysis. Adv. Simul..

[31] Sarmah M., Neelima A., Singh H.R. (2023). Survey of methods and principles in three-dimensional reconstruction from two-dimensional medical images. Vis. Comput. Ind. Biomed. Art.

[32] Bhuskute H., Shende P., Prabhakar B. (2022). 3D printed personalized medicine for cancer: Applications for betterment of diagnosis, prognosis and treatment. AAPS PharmSciTech.

[33] Europe A. (2024). Artec 3D Portable Scanners. https://www.artec3d.com.

[34] Haleem A., Javaid M., Singh R.P., Rab S., Suman R., Kumar L., Khan I.H. (2022). Exploring the potential of 3D scanning in Industry 4.0: An overview. Int. J. Cogn. Comput. Eng..

[35] Rieke-Zapp D., Royo S. (2017). Structured light 3D scanning. Digital Techniques for Documenting and Preserving Cultural Heritage.

[36] Scaniverse Review: Free 3D Laser Scans with Your iPhone—Structural Basics—Structuralbasics.com. https://www.structuralbasics.com/scaniverse-review/.

[37] Goesele M., Snavely N., Curless B., Hoppe H., Seitz S.M. (2007). Multi-view stereo for community photo collections. Proceedings of the 2007 IEEE 11th International Conference on Computer Vision.

[38] Brière-Côté A., Rivest L., Maranzana R. (2012). Comparing 3D CAD models: Uses, methods, tools and perspectives. Comput. Aided Des. Appl..

[39] Samavati T., Soryani M. (2023). Deep learning-based 3D reconstruction: A survey. Artif. Intell. Rev..

[40] Tachella J., Altmann Y., Mellado N., McCarthy A., Tobin R., Buller G.S., Tourneret J.Y., McLaughlin S. (2019). Real-time 3D reconstruction from single-photon lidar data using plug-and-play point cloud denoisers. Nat. Commun..

[41] Yin X., He J., Cheng Z. (2025). Efficient and lightweight 3D building reconstruction from drone imagery using sparse line and point clouds. Virtual Real. Intell. Hardw..

[42] Sitzmann V., Martel J., Bergman A., Lindell D., Wetzstein G. (2020). Implicit neural representations with periodic activation functions. Adv. Neural Inf. Process. Syst..

[43] Mildenhall B., Srinivasan P.P., Tancik M., Barron J.T., Ramamoorthi R., Ng R. (2021). Nerf: Representing scenes as neural radiance fields for view synthesis. Commun. ACM.

[44] Kerbl B., Kopanas G., Leimkühler T., Drettakis G. (2023). 3d gaussian splatting for real-time radiance field rendering. ACM Trans. Graph..

[45] Wu T., Yuan Y.J., Zhang L.X., Yang J., Cao Y.P., Yan L.Q., Gao L. (2024). Recent advances in 3d gaussian splatting. Comput. Vis. Media.

[46] Fei B., Xu J., Zhang R., Zhou Q., Yang W., He Y. (2024). 3d gaussian splatting as new era: A survey. IEEE Trans. Vis. Comput. Graph..

[47] Schonberger J.L., Frahm J.M. (2016). Structure-from-motion revisited. Proceedings of the IEEE Conference on Computer Vision and Pattern Recognition.

[48] Chang A.X., Funkhouser T., Guibas L., Hanrahan P., Huang Q., Li Z., Savarese S., Savva M., Song S., Su H. (2015). Shapenet: An information-rich 3d model repository. arXiv.

[49] Wang X., Li P. (2020). Extraction of urban building damage using spectral, height and corner information from VHR satellite images and airborne LiDAR data. ISPRS J. Photogramm. Remote Sens..

[50] Altuntas C. (2023). Review of Scanning and Pixel Array-Based LiDAR Point-Cloud Measurement Techniques to Capture 3D Shape or Motion. Appl. Sci..

[51] Xu J., Xi N., Zhang C., Zhao J., Gao B., Shi Q. (2011). Rapid 3D surface profile measurement of industrial parts using two-level structured light patterns. Opt. Lasers Eng..

[52] Weinmann M., Schwartz C., Ruiters R., Klein R. (2011). A multi-camera, multi-projector super-resolution framework for structured light. Proceedings of the 2011 International Conference on 3D Imaging, Modeling, Processing, Visualization and Transmission.

[53] Geiger A., Ziegler J., Stiller C. (2011). Stereoscan: Dense 3d reconstruction in real-time. Proceedings of the 2011 IEEE Intelligent Vehicles Symposium (IV).

[54] Furukawa Y., Hernández C. (2015). Multi-view stereo: A tutorial. Found. Trends Comput. Graph. Vis..

[55] Khot T., Agrawal S., Tulsiani S., Mertz C., Lucey S., Hebert M. (2019). Learning unsupervised multi-view stereopsis via robust photometric consistency. arXiv.

[56] Yu A., Ye V., Tancik M., Kanazawa A. pixelnerf: Neural radiance fields from one or few images. Proceedings of the IEEE/CVF Conference on Computer Vision and Pattern Recognition.

[57] Barron J.T., Mildenhall B., Verbin D., Srinivasan P.P., Hedman P. Mip-nerf 360: Unbounded anti-aliased neural radiance fields. Proceedings of the IEEE/CVF Conference on Computer Vision and Pattern Recognition.

[58] Li Z., Müller T., Evans A., Taylor R.H., Unberath M., Liu M.Y., Lin C.H. Neuralangelo: High-fidelity neural surface reconstruction. Proceedings of the IEEE/CVF Conference on Computer Vision and Pattern Recognition.

[59] Garbin S.J., Kowalski M., Johnson M., Shotton J., Valentin J. Fastnerf: High-fidelity neural rendering at 200 fps. Proceedings of the IEEE/CVF International Conference on Computer Vision.

[60] Deng K., Liu A., Zhu J.Y., Ramanan D. Depth-supervised nerf: Fewer views and faster training for free. Proceedings of the IEEE/CVF Conference on Computer Vision and Pattern Recognition.

[61] Kwak M.S., Song J., Kim S. (2023). GeCoNeRF: Few-shot Neural Radiance Fields via Geometric Consistency. Proceedings of the International Conference on Machine Learning.

[62] Takikawa T., Litalien J., Yin K., Kreis K., Loop C., Nowrouzezahrai D., Jacobson A., McGuire M., Fidler S. Neural geometric level of detail: Real-time rendering with implicit 3d shapes. Proceedings of the IEEE/CVF Conference on Computer Vision and Pattern Recognition.

[63] Hu T., Liu S., Chen Y., Shen T., Jia J. Efficientnerf efficient neural radiance fields. Proceedings of the IEEE/CVF Conference on Computer Vision and Pattern Recognition.

[64] Yuan Y.J., Sun Y.T., Lai Y.K., Ma Y., Jia R., Gao L. Nerf-editing: Geometry editing of neural radiance fields. Proceedings of the IEEE/CVF Conference on Computer Vision and Pattern Recognition.

[65] Ye V., Li R., Kerr J., Turkulainen M., Yi B., Pan Z., Seiskari O., Ye J., Hu J., Tancik M. (2025). gsplat: An open-source library for Gaussian splatting. J. Mach. Learn. Res..

[66] Guédon A., Lepetit V. Sugar: Surface-aligned gaussian splatting for efficient 3d mesh reconstruction and high-quality mesh rendering. Proceedings of the IEEE/CVF Conference on Computer Vision and Pattern Recognition.

[67] Zhang B., Fang C., Shrestha R., Liang Y., Long X., Tan P. (2024). Rade-gs: Rasterizing depth in gaussian splatting. arXiv.

[68] Turkulainen M., Ren X., Melekhov I., Seiskari O., Rahtu E., Kannala J. (2025). Dn-splatter: Depth and normal priors for gaussian splatting and meshing. Proceedings of the 2025 IEEE/CVF Winter Conference on Applications of Computer Vision (WACV).

[69] Chung J., Oh J., Lee K.M. Depth-regularized optimization for 3d gaussian splatting in few-shot images. Proceedings of the IEEE/CVF Conference on Computer Vision and Pattern Recognition.

[70] Thai A., Peng S., Genova K., Guibas L., Funkhouser T. Splattalk: 3d vqa with gaussian splatting. Proceedings of the IEEE/CVF International Conference on Computer Vision.

[71] Jung J., Han J., An H., Kang J., Park S., Kim S. (2024). Relaxing accurate initialization constraint for 3d gaussian splatting. arXiv.

[72] Sauvalle B., de La Fortelle A. Autoencoder-based background reconstruction and foreground segmentation with background noise estimation. Proceedings of the IEEE/CVF Winter Conference on Applications of Computer Vision.

[73] Rublee E., Rabaud V., Konolige K., Bradski G. (2011). ORB: An efficient alternative to SIFT or SURF. Proceedings of the 2011 International Conference on Computer Vision.

[74] Fischler M.A., Bolles R.C. (1981). Random sample consensus: A paradigm for model fitting with applications to image analysis and automated cartography. Commun. ACM.

[75] Lu X.X. (2018). A review of solutions for perspective-n-point problem in camera pose estimation. J. Phys. Conf. Ser..

[76] Mustaniemi J., Kannala J., Särkkä S., Matas J., Heikkilä J. (2017). Inertial-based scale estimation for structure from motion on mobile devices. Proceedings of the 2017 IEEE/RSJ International Conference on Intelligent Robots and Systems (IROS).

[77] Wkeglarczyk S. (2018). Kernel density estimation and its application. ITM Web Conf..

[78] Mullen T. (2011). Mastering Blender.

[79] Padberg T., Heikkonen J., Kanth R. (2024). Study on Stereo AI Based Zed-2i Camera. Proceedings of the International Conference on Information Technology & Systems.

[80] Qin X., Dai H., Hu X., Fan D.P., Shao L., Van Gool L. (2022). Highly accurate dichotomous image segmentation. Proceedings of the European Conference on Computer Vision.

[81] Setiadi D.R.I.M. (2021). PSNR vs. SSIM: Imperceptibility quality assessment for image steganography. Multimed. Tools Appl..

